# Editorial: Welcome to *APL Bioengineering*

**DOI:** 10.1063/1.5007077

**Published:** 2017-10-09

**Authors:** Justin Cooper-White

**Affiliations:** The Australian Institute for Bioengineering and Nanotechnology, University of Queensland, Brisbane, Queensland, St Lucia, 4072 QLD, Australia

**Figure:**
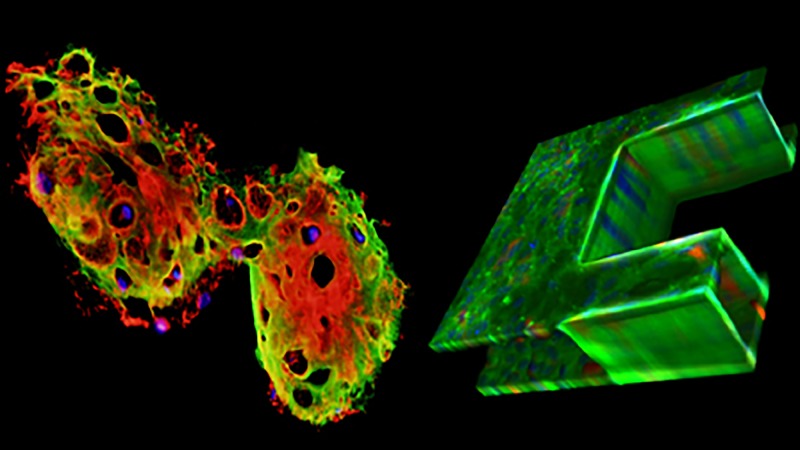


Welcome to the first issue of *APL Bioengineering*!

The bioengineering research community is now a substantial force, in terms of both number of researchers and contributions to society. The field exposes a new understanding of the complexity of biological systems and enhances our ability to measure, manipulate, and engineer them for therapeutic benefit. In response to clear health, economic, and social benefits, governments and industries across the globe are actively supporting research advancements in this field.

Global industry sectors rely on and are supported by bioengineering principles and discoveries; more broadly, bioengineering has changed the way we perceive the future of medicine and human health. It is one of the most rapidly developing multidisciplinary engineering fields. With applied physics at its core, the bioengineering community can clearly benefit from a new journal that takes a multidisciplinary approach to the latest scientific breakthroughs and next-generation innovations in the field—*APL Bioengineering*. The global context and industrial significance of bioengineering require an open access format to ensure that all published research has the widest possible dissemination to the community's many intersecting disciplines.

In the tradition of the editorial excellence we have come to expect from AIP Publishing, *APL Bioengineering* will share the same high level of scientific and academic rigor as *Applied Physics Letters* (APL) and sister journals *APL Materials* and *APL Photonics*. *APL Bioengineering* will be *the* place for bioengineers, biomedical engineers and scientists, chemical engineers, applied mathematicians, and physicists to publish high-impact papers specific to understanding, characterizing, and manipulating the physics of engineered systems with proven applications in the bioprocessing, gene therapy, cellular therapies, and regenerative medicine arenas. The advancement of medicine or enhancement of human health is critical to impact, and *APL Bioengineering* will require this evidence of translation (or a minimal barrier to such) of all its contributions.

The international team of Associate Editors has been appointed for their outstanding contributions as researchers across multiple disciplines and their keen insight into bioengineering's future impact on the advancement of science. They are all well known to the community and will play a key role in shaping the editorial coverage of *APL Bioengineering*. Our Editorial Advisory Board comprises world-leading researchers and will offer an even broader community perspective to help guide and support the journal now and in the future.

The scope of *APL Bioengineering* encompasses a wide range of topics within the field, from bioprinting to bioimaging, biophysics to tissue engineering, and drug delivery to gene therapy. The journal welcomes submissions that contain fundamental research that advances the understanding of physics and engineering of biological systems, or translational research that applies physics and engineering to significantly advance medicine or human health.

Alongside primary research, we will be inviting authors to submit Perspectives and Reviews. Perspectives give a forward-looking view into the challenges in a specific subject area, and Reviews provide the community with a succinct overview of a focused bioengineering topic. Beginning in 2018, we will also begin publishing Special Topics that will present Articles, Perspectives, and Reviews on emerging, timely topics in bioengineering. The first Special Topic, on the Bioengineering of Cancer, guest edited by Associate Editor Adam Engler and Editorial Advisory Board member Dennis Discher, will be open for submissions this Fall.

This issue of *APL Bioengineering* is the first of many issues that will enable bioengineering research to continue to grow and contribute to our society in ways yet to be imagined. Thanks to the authors who have trusted us with their manuscripts since we opened for submissions, the reviewers who have embraced the scope and editorial standards of the journal, and the Associate Editors for their careful consideration of every single paper. I look forward to continuing to work with the Editorial team and the growing community of *APL Bioengineering* authors, reviewers, and readers who are eager to be a part of something new. We look forward to publishing your latest discoveries!

